# Tissue biochemical diversity of 20 gooseberry cultivars and the effect of ethylene supplementation on postharvest life

**DOI:** 10.1016/j.postharvbio.2016.02.008

**Published:** 2016-07

**Authors:** Maria Anastasiadi, Paul M. Mwangi, José J. Ordaz-Ortiz, Sally P. Redfern, Mark Berry, Monique S.J. Simmonds, Leon A. Terry

**Affiliations:** a Plant Science Laboratory, Cranfield University, Bedfordshire, MK43 0AL, UK; b Unilever R&D Colworth, Sharnbrook, Bedfordshire, MK44 1LQ, UK; c Royal Botanic Gardens, Kew, Surrey, TW9 3AB, UK

**Keywords:** *Ribes uva-crispa*, Ascorbic acid, QToF/MS, Ethylene, Storage, Bioactive life

## Abstract

The European gooseberry (*Ribes uva-crispa*) is still an understudied crop with limited data available on its biochemical profile and postharvest life. A variety of polyphenols were detected in the skin and flesh of 20 gooseberry cvs, representing mainly flavonol glycosides, anthocyanins and flavan-3-ols. In contrast, gooseberry seeds were for the first time characterised by the presence of considerable amounts of hydroxycinnamic acid glycosides tentatively identified by UPLC-QToF/MS. All cvs examined represented a good source of vitamin C while being low in sugar. Furthermore, the postharvest stability of bioactives was explored by supplementation of exogenous ethylene in air at 5 °C. Results suggest a low sensitivity of gooseberries to ethylene. The overall quality of gooseberries remained stable over two weeks, showing potential for extended bioactive life.

## Highlights

• Biochemical profile of English grown gooseberries and differences between tissues characterised.
• Effects of exogenous ethylene application prior to storage were investigated.
• ‘Scotch Red Rough’ and ‘Heart of Oak’ where some of the cvs with the highest phytochemical potency.
• Total anthocyanin content increased with storage time.
• Exogenous ethylene did not negatively impact the quality of gooseberries

## 1 Introduction

Gooseberries are deciduous shrubs being members of the *Grossulariaceae* family and genus *Ribes* like blackcurrants, redcurrants, whitecurrants and jostaberries (Bordonaba and Terry, 2011). The genus is comprised of more than 150 diverse species with currants and gooseberries being the most popular (Barney and Hummer, 2005). *Ribes uva-crispa* L. (synonym *Ribes grossularia* L.) is a European species and the most prevalent species among the gooseberries found across the world. It is native to United Kingdom, Caucasus Mountains and North West Africa (Barney and Hummer, 2005). The size of gooseberries varies as does their skin colour ranging from green to pink, red, purple, white, and yellow (Hummer and Dale, 2010). The commercial value of gooseberries is limited at present, mainly due to low demand and high cost of production especially during harvesting (Barney and Hummer, 2005, Dale, 2000). Other influencing factors include prevalence of crop diseases such as powdery mildew (Barney and Hummer, 2005) and lack of high yielding cvs (Pluta et al., 2010).

In recent years there is a rising trend in domestic cultivation of *Ribes* berries both in Europe and other regions (Barney and Hummer, 2005, Mitchell et al., 2011). Reasons for the increasing interest include small agricultural requirements, resistance to cold winters and the development of improved cvs with better disease resistance, colour, flavour and yield. Quality of gooseberries is primarily based on its visual, textural, organoleptic and nutritional characteristics (Terry et al., 2009). The later attribute has attracted considerable interest over the years, as some bioactive components in berries have been associated with potential health-promoting properties (Folmer et al., 2014, Wang and Stoner, 2008).

Despite the amount of information on the qualitative and quantitative content of bioactives in berries, the nutritional quality of gooseberries has not yet been sufficiently explored due to limited commercial interest. Only a few gooseberry cvs have been studied thus far in which, polyphenols have been extracted from the fruit as a whole (Jordheim et al., 2007, Määttä-Riihinen et al., 2004, Pantelidis et al., 2007, Wu et al., 2004). There is limited information about the spatial contribution across tissues in the final polyphenolic content of gooseberries. The main phenolic compounds reported in gooseberries include anthocyanins (Jordheim et al., 2007, Määttä-Riihinen et al., 2004, Pantelidis et al., 2007, Wu et al., 2004), flavonol glycosides and proanthocyanins (Chiang et al., 2013, Häkkinen et al., 1999a, Mikulic-Petkovsek et al., 2012a, Russell et al., 2009).

The limited commercial value of gooseberries is also depicted in the scarce data available on the postharvest life of gooseberries and the stability of their presumed bioactive components during storage. At present, only a few reports exist on the physical, physiological and biochemical changes occurring in gooseberries during different storage conditions (Harb and Streif, 2004, Kampuse et al., 2015, Muizniece-Brasava et al., 2015). In addition, a better understanding is needed on the role of ethylene in the postharvest life of gooseberries. Gooseberries are classified as non-climacteric fruits, although they are able to produce ethylene in low amounts (0.035–0.35 ng kg^−1^ s^−1^ at 20 °C) (Cantwell, 2002, Thompson, 2002). The role of ethylene on *Ribes* berries, however, has not been thoroughly investigated with reports often contradictory regarding their sensitivity (Cantwell, 2002, McKay and Van Eck, 2006).

The objective of this study was thus two-fold: to explore the biochemical profile of different tissues of a wide selection of gooseberry varieties grown in the UK, and elucidate postharvest changes in biochemistry and quality characteristics for two gooseberry varieties held for 15 days at low temperature with or without application of exogenous ethylene.

## 2 Material and methods

### 2.1 Chemicals

All HPLC and LC–MS grade solvents were obtained from Fisher Scientific (Loughborough UK). (+)-catechin, (−)-epicatechin, procyanidin B1, procyanidin B2, neochlorogenic acid, caffeic acid, sinapic acid, *p*-coumaric acid, quercetin-rutinoside, quercetin-glucoside, isorhamnetin-glucoside, isorhamnetin-rutinoside were purchased from Sigma–Aldrich (Dorset, UK). Cyanidin-3-glucoside and cyanidin-3-rutinoside, were purchased from Extrasynthese (Genay Cedex, France). Metaphosphoric acid (Bioxtra ≥ 33.5%), l-ascorbic acid and d-fructose were obtained from Sigma–Aldrich (Dorset, UK). d-glucose and sucrose, were purchased from Fisher Scientific (Loughborough UK).

### 2.2 Plant material and sample preparation

**Fig. 1:**
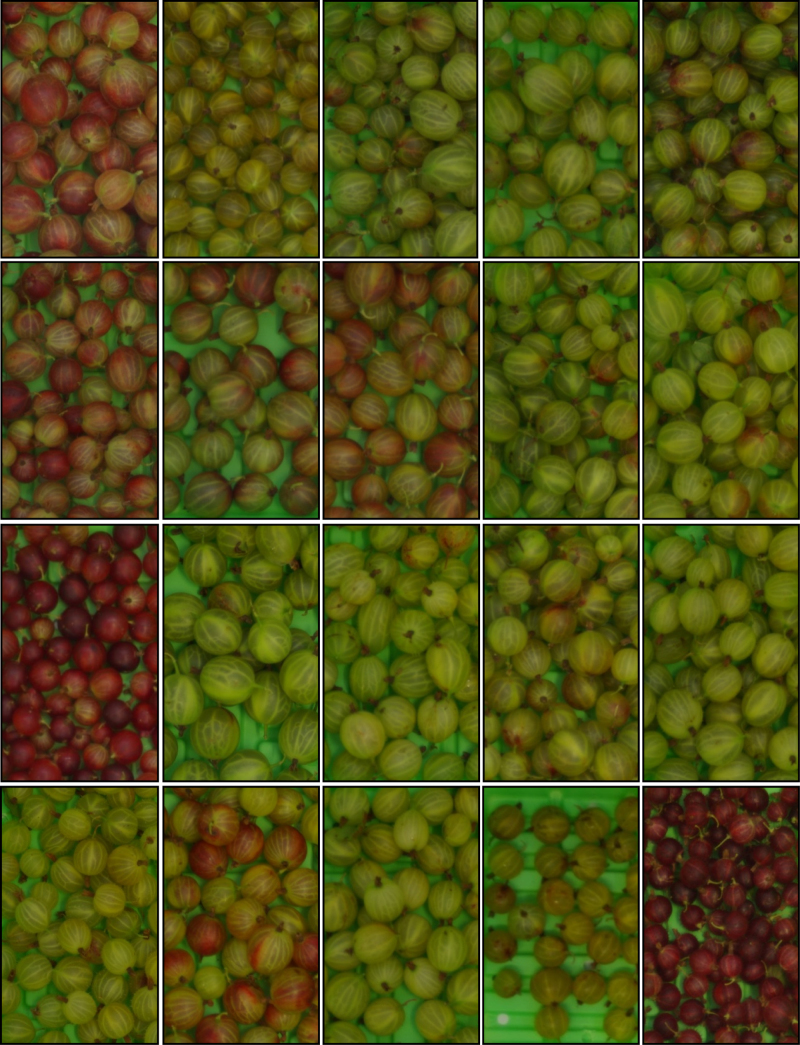
Phenotypic differences of 20 gooseberry cvs, harvested from the National Fruit Collection in 2012. From left to right: ‘Careless 3024′, ‘Guy seedling’, ‘Jolly Amylers’, ‘Jubilee Careless’, ‘Heart of Oak’, ‘Victoria’, ‘Ajax’, ‘Goutray’s Earliest’, ‘Rubuste Nool’, ‘Lord Elco (Scotland)’, ‘May Dulle’, ‘White Eagle’, ‘Careless (Kent)’, ‘Nailer’, ‘Mitre’, ‘Cousen's Seedling’, ‘Lord Audley’, ‘Careless VT 512′, ‘Bedford Yellow’, ‘Scotch Red Rough’.

Gooseberry fruits from 20 cvs of *R. uva-crispa* were obtained at optimum maturity, from The National Fruit Collection (Brogdale, Kent, UK) on the 13th of July 2012 for biochemical analysis (Fig. 1). Based on the biochemical results obtained, two cvs ‘Careless (Kent)’ and ‘Scotch Red Rough’ were selected and harvested again the following year (12th of July 2013) for the purposes of the postharvest trial. Approximately 100–200 berries were harvested from two plants per cv at optimum maturity stage and transported to Cranfield University in cool boxes within 2 h from collection and immediately snap-frozen in liquid nitrogen. The samples were divided into triplicates and stored at −80 °C before analysis. Each sample was further divided into two subsamples. Half of the material (approximately 100 g) was freeze-dried, the seeds were manually removed and the berries were ground into a fine powder for the extraction of phenolics and sugars. The second subsample was kept fresh frozen and powdered in a mortar grinder (RM 200, Retsch Ltd., Derbyshire, UK) with liquid N_2_ and used for the extraction of ascorbic acid to avoid potential degradation of ascorbic acid during the freeze-drying process.

### 2.3 Postharvest trial

Samples from the two selected gooseberry cvs (‘Scotch Red Rough’ and ‘Careless (Kent)’) were transferred inside polystyrene boxes with ice-packs, from The National Fruit Collection to Cranfield within 3 h from harvest. Upon arrival at Cranfield, gooseberries from each cv were split into two batches. The four batches of gooseberries were placed in plastic stackable crates inside water sealed, air-tight polypropylene chambers (88 cm × 59 cm × 59 cm) fitted with two 8 × 8 cm electric fans (Nidec beta SL, RS Components Ltd., Northants, UK) to circulate the ethylene gas during treatment. Two boxes per cv were injected with 11.69 μg L^−1^ of standard ethylene gas (100% ethylene; SIP analytical) and the other two boxes were used as control samples (untreated). Temperature of the storage room was set at 5 °C and the treatment time was 24 h. The concentration of the ethylene gas in the chambers was confirmed after 30 min of injection and after 24 h of storage by withdrawing air from the chambers (including controls), using a tapped 20 mL plastic syringe. The headspace of the sampled air was injected into a gas chromatograph (GC—Model 8340, DP800 integrator, Carlos Erba Instruments, Herts, UK, analytical column, Porapak) fitted with flame ionisation detector (FID) and ethylene gas present quantified.

After the treatment was completed (24 h), every batch was split into three replicates and stored in ventilated propylene (15 cm × 22 cm × 8 cm) containers. The containers had an inlet and outlet that provided ventilation to the fruits by way of pumping air into the boxes at a flow rate of about 3.33 mL s^−1^ from a flowmeter controlled unit attached to a ICA 6000 (International Controlled Atmosphere Ltd., Kent, UK). The RH inside the boxes was maintained at 80 ± 10% by placing a beaker of water inside the containers. The RH and temperature in the boxes was continuously monitored using Tinytag Ultra 2 TGU-4500 data loggers (−95% RH, −25 to 85 °C, Gemini Data Loggers Ltd., West Sussex, UK).

#### 2.3.1 Sampling during storage period

Upon arrival (Day 0), five gooseberries were randomly selected from each cv in triplicate. After the treatment (Day 1), five gooseberries were sampled randomly from each box (3 boxes per treatment). Sampling was thereafter repeated at regular intervals (Day 4, Day 7, Day 11, Day 13, Day 15). All samples were subjected to colour and ethylene production measurements before being snap-frozen in liquid nitrogen. The snap-frozen berries were stored at −80 °C until analysis. Prior to extraction the plant material was freeze-dried and powdered as before and analysed for individual soluble sugars and phenolics. Water content of gooseberries was calculated by recording the weight of all samples before and after freeze-drying.

### 2.4 HPLC-ELSD analysis of non-structural carbohydrates

Extraction of non-structural carbohydrates was performed according to a previous method with slight modifications (Terry et al., 2007). Prior to analysis, the sugars extracts were diluted (1:9, v/v) with HPLC grade water and injected into an Agilent 1200 HPLC fitted with a prevail carbohydrate ES 5 μm size of 250 nm × 4.6 mm diameter and a guard column of the same type. The mobile phase consisted of solvent A (water) and solvent B (acetonitrile) and the elution gradient was as follows: 0–15 min, 80–50% B, 15–20 min, 50–20% B, 20–25 min, 80% B. The eluted compounds were detected by evaporative light scattering detector (ELSD) and quantification was based on external calibration curves of commercial standards.

### 2.5 Phenolic compounds

#### 2.5.1 Extraction of phenolic compounds

Extraction of phenolic compounds from berries and seeds was performed according to a previous method with slight modifications (Giné Bordonaba and Terry, 2008). Freeze–dried berry powder (150 mg) and freeze–dried seed powder (50 mg) were extracted with 3 mL and 1 mL, respectively, of methanol/water/HCl (70:29.5:0.5 v/v) in a shaking water bath at 20 °C for 1.5 h. The samples were vortexed every 10 min to re-suspend the solids. The extracts were subsequently filtered with 0.20 μm nylon filters and stored at −40 °C before analysis.

#### 2.5.2 HPLC-DAD analysis of phenolic compounds and fraction collection

Analysis of flavan-3-ols, dimeric procyanidins, flavonol glycosides, anthocyanins and neochlorogenic acid in gooseberry extracts (flesh/skin) was performed on a Agilent 1200 HPLC system with a diode array detector (DAD) detector, equipped with a quaternary pump, a fraction collector, a thermostated column compartment operating at 30 °C and a cooled autosampler set at 6 °C. Separation was achieved with an Eclispse XDB-C18 column (150 mm × 4.6 mm; 5 μm, Agilent Technologies) and a 1 mm OPTI-Guard column (mobile phases consisted of 3% formic acid in water) (solvent A) and acetonitrile (solvent B) and the flow rate was 17 μL s^−1^. The elution gradient was as follows: 0–5 min, 3–5% B, 5–10 min; 5–10% B, 10–20 min, 10–30% B; 20–25 min, 30–60% B; 25–26 min, 60–100% B, 26–30 min, 100% B, followed by 5 min re-equilibration time. The DAD detector was operating at 4 different wavelengths, 280 nm, 320 nm, 360 nm and 520 nm. Quantification was based on external calibration curves of commercial standards. Six anthocyanins detected at 520 nm for which commercial standards were not available, were quantified based on the calibration curve obtained for cyanidin-3-glucoside. Furthermore the fraction collector was used to isolate those peaks and subject them to LC/MS analysis for further elucidation.

#### 2.5.3 UPLC/QToF/MS analysis of unknown anthocyanins

The six fractions isolated at 520 nm with UV spectra similar to the anthocyanin standards, were further analysed with an Agilent 1290 infinity UPLC system, comprised of a binary pump with a jet weaver V35 mixer, a thermostated column, set at 30 °C, and a cooled autosampler set at 6 °C. The UPLC system was coupled with an Agilent 6540 Ultra High Definition (UHD) Accurate Mass Q-TOF–MS system (Agilent Technologies) equipped with an electrospray ionization source (Agilent Dual Jet Stream). The fractions were evaporated using a miVac centrifugal vacuum concentrator (Genevac) and reconstituted with 100 μL 0.1% formic acid in water and 5 μL were injected into a ZORBAX SB-C18 Rapid resolution, HD 2.1 × 150 mm, 1.8 μm column (Agilent Technologies). Mobile phases consisted of 0.1% formic acid in water (solvent A) and 0.05% formic acid in acetonitrite (solvent B) and the flow was 6.67 μL s^−1^. The elution gradient was as follows: 0.5–6.5 min, 5% B, 6.5–8 min; 5–15% B, 8–15.5 min, 15–20% B; 15.5–15.55 min, 20–45% B; 15.55–17.55 min, 45–100% B, 17.55–17.60 min, 100–5% B; 17.60–20.0 min, 5% B. Source conditions for the MS were set as follows: nebulizer gas (N_2_) temperature 150 °C, at a flow rate of 0.13 L s^−1^ sheath gas (N_2_) temperature 400 °C, at a of flow rate of 0.18 L s^−1^ and fragmentor voltage 165 V. MS spectra acquired in full scan, positive ionisation mode in the *m/z* 50–1500 range.

MS/MS experiments were subsequently carried in positive mode; to obtain the daughter ion spectrum of the selected precursor ions. Product ions were produced by collision-induced dissociation using targeted MS/MS experiments, at three different collision energies (10, 20, 40 eV) with an isolation window of 4 *m/z* (medium) for all compounds. The LC-MS system and data acquisition were controlled by an Agilent MassHunter Data Acquisition software B.04.00.

#### 2.5.4 UPLC/QToF/MS analysis of hydroxycinnamic acid derivatives

The gooseberry extracts obtained from different tissues (skin/fresh and seeds) were further analysed with the Agilent 1290 infinity UPLC system and the Agilent 6540 UHD Accurate Mass Q-TOF-MS system described above. All extracts were further diluted (1:4) with 0.1% formic acid in water and 5 μL were injected into a ZORBAX SB-C18 column (Rapid resolution, HD 2.1 × 150 mm, 1.8 μm; Agilent Technologies) with the chromatographic method described in Section 2.5.3. Commercial standards of different phenolic compound groups were used as reference compounds (hydroxycinnamic acids, flavonols, flavan-3-ols, dimeric procyanidins).

MS/MS experiments in positive mode were subsequently carried on selected precursor ions to assist with compound identification. Product ions were produced by collision-induced dissociation using targeted MS/MS experiments, at three different collision energies (10, 20, 40 eV) with an isolation window of 4 *m/z* (medium) for all compounds.

Quantification of the hydroxycinnamic acid derivatives identified with the above method was based on external calibration curves of the corresponding aglycons (*p*-coumaric acid, caffeic acid, ferulic acid, sinapic acid).

### 2.6 Extraction and quantification of total ascorbic acid

The total ascorbic acid of the 20 gooseberry cvs harvested in 2012, was determined as total vitamin C using a previously described method with slight modifications (Planchon et al., 2004). The method determined the total vitamin C in the gooseberries as the sum of ascorbic acid and its oxidised form dehydroascorbic acid. Briefly, 500 mg of fresh frozen gooseberries were mixed with 2.5 mL of degassed metaphosphoric acid solution (0.01 M) and the mixture was extracted in a shaking water bath at 25 °C for 10 min. The extract was filtered through 0.20 μm nylon filters and immediately injected in the HPLC. Ascorbic acid was monitored at 248 nm and the total vitamin C was quantified using an external calibration curve of l-ascorbic acid. The assay was performed in triplicate.

### 2.7 Colour measurement

The colour of gooseberries was assessed using an 8 mm aperture handheld tristimulus colourimeter (Minolta CR- 400, Osaka, Japan). The mean colour space coordinates of Lightness (L*), Chroma (C*) and Hue angle (*H*°) were taken from three positions on each gooseberry for every sampling point.

### 2.8 Ethylene production measurement

The ability of the gooseberries to produce ethylene gas was measured with an ETD-300 Ethylene Detector system (Sensor Sense BV, Nijmegen, The Netherlands). In order to determine the amount of ethylene in the cuvettes after 1 h incubation, air was pumped at a continuous flow rate of 1.1 μL s^−1^. The air from the cuvettes was passed through CO_2_ and water scrubbers. Measurements from every cuvette were made for a period of 12 min and expressed in ng kg^−1^ s^−1^. The equipment was equilibrated before measurement by measuring an empty cuvette for 1 h. A baseline measurement of 3 min was maintained between the samples to eliminate cross contamination.

### 2.9 Statistical analysis

Analysis of variance (one-way and two-way ANOVA) was performed using Genstat for Windows, Version 14 (VSN International Ltd., Herts., UK). The differences between means of data were compared through Least Significance Difference (LSD) and they were considered to be statistically significant at the 95% confidence level *(p* *≤* 0.05). Logarithmic transformations were employed where needed in order to ensure the assumption of equal variability.

## 3 Results and discussion

### 3.1 Phenolic composition of gooseberries

#### 3.1.1 Characterization and quantification of non-coloured compounds in skin/flesh

Gooseberries (flesh/skin) contained a variety of phenolic compounds including flavonol glycosides, flavan-3-ols, dimeric procyanidins, and hydroxycinnamic acid derivatives (Table 1, Table 2). The major class of polyphenols present in all cvs was flavonol glycosides with the rutinosides of quercetin and isorhamnetin being the predominant glycosides. Quercetin and isorhamnetin glucoside on the other hand were found in much lower amounts. Mikulic-Petkovsek et al. (2012a) also observed that quercetins in gooseberries existed as glucosides and rutinosides and that these are the main form in which they exist in berries from *Glossulariaceae* family. The same authors have also reported high concentration of isorhamnetin-3-rutinoside in gooseberries (73.9 mg kg^−1^ in red and 48.3 mg kg^−1^ in white gooseberries for FW) when compared to other common berries such as bilberry, blackcurrant and strawberry. Similarly, Määttä-Riihinen et al. (2004) have also reported high concentration of isorhamnetin glycosides in gooseberries. Our results are in good agreement with these previous reports, although the concentration range of quercetin glycosides among the 20 gooseberry cvs analysed within this study were higher than those previously reported (Häkkinen et al., 1999a, Häkkinen et al., 1999b, Määttä-Riihinen et al., 2004, Mikulic-Petkovsek et al., 2012a). These differences could reflect that, to date, only a limited number of gooseberry cvs have been studied. However, the overall trend is in line with Määttä-Riihinen et al. (2004) observation that quercetins are high in berries from *Glossulariaceae* family. Other flavonol glycosides reported in gooseberries, include glycosides of myricetin and kaempferol, typically present in the form of rutinoside representing minor constituents of the flavonol content of gooseberries (Määttä-Riihinen et al., 2004, Mikulic-Petkovsek et al., 2012a). Furthermore Mikulic-Petkovsek et al. (2012a), identified the presence of small amounts of lacitrin and syringetin rutinoside in a white gooseberry cv and other berry species.

**Table 1:** Flavonoid composition (mg kg^−1^) of the flesh/skin of 20 gooseberry cvs on a fresh weight (FW) and dry weight (DW) basis (values inside brackets).^a^

Cultivar	(+)-Catechin		(−)-Epicatechin		ProcB1^b^		ProcB^c^		Q-3-rut^d^		Q-3-gluc^e^		Isorh-3-rut^f^		Isorh-3-gluc^g^	
Scotch Red Rough	20.1	(112.8)	4.2	(23.7)	8.8	(49.5)	2.5	(14.3)	102.5	(767.9)	13.7	(102.7)	81.0	(606.4)	2.7	(20.4)
Lord Audley	26.5	(224.8)	2.2	(18.7)	9.3	(79.2)	2.4	(20.2)	78.2	(585.6)	7.0	(52.8)	38.3	(287.2)	1.6	(12.3)
Heart of Oak	44.3	(264.5)	2.4	(13.9)	12.9	(75.4)	2.3	(13.6)	55.3	(414.1)	7.1	(52.8)	26.8	(200.4)	1.2	(8.7)
Nailer	37.0	(219.3)	2.2	(13.0)	13.1	(70.3)	2.4	(14.4)	49.9	(373.8)	4.8	(35.9)	29.3	(219.1)	1.4	(10.1)
Jolly Amylers	56.5	(364.0)	2.4	(15.6)	16.4	(105.4)	2.3	(15.0)	25.8	(192.9)	3.4	(25.3)	24.0	(179.6)	1.7	(12.7)
Bedford Yellow	46.6	(326.2)	2.7	(18.5)	18.4	(128.5)	4.5	(31.2)	37.5	(280.6)	2.9	(21.9)	13.9	(104.0)	0.9	(6.6)
Cousen’s Seedling	14.0	(112.6)	2.0	(16.1)	10.3	(82.5)	3.9	(31.4)	44.7	(334.5)	5.0	(37.3)	40.6	(304.2)	2.5	(18.6)
Careless (Kent)	10.9	(83.2)	1.0	(7.4)	7.0	(53.7)	1.0	(7.8)	43.1	(322.8)	5.3	(39.9)	28.7	(215.0)	2.3	(17.0)
Victoria	34.6	(285.2)	2.3	(18.8)	12.5	(102.2)	2.6	(21.0)	25.7	(192.4)	3.0	(22.5)	16.4	(123.2)	0.5	(3.9)
White Eagle	32.6	(209.6)	2.1	(13.4)	12.4	(79.3)	2.6	(16.8)	16.2	(121.4)	3.3	(25.1)	24.5	(183.2)	2.2	(16.6)
Jubilee Careless	28.5	(192.9)	1.7	(11.6)	10.5	(71.2)	1.6	(10.9)	24.6	(184.1)	2.5	(18.6)	19.5	(145.9)	1.2	(9.3)
Goutray’s Earliest	29.6	(258.0)	3.0	(25.8)	10.4	(90.9)	3.7	(32.1)	20.6	(154.4)	3.0	(22.4)	18.1	(135.2)	1.4	(10.6)
May Dulle	15.1	(106.9)	2.6	(18.5)	9.7	(68.6)	1.9	(13.7)	30.6	(229.5)	5.8	(43.5)	21.4	(160.0)	1.5	(11.0)
Mitre	17.8	(126.5)	1.3	(09.5)	8.2	(58.2)	1.2	(8.9)	33.9	(254.0)	3.7	(27.6)	18.8	(141.1)	1.2	(8.9)
Lord Elco	40.1	(320.3)	1.4	(10.9)	13.0	(103.3)	1.7	(13.6)	9.6	(71.6)	0.6	(4.1)	18.3	(137.3)	1.4	(10.9)
Rubuste Nool	30.1	(206.1)	1.6	(10.9)	11.1	(76.0)	1.5	(10.0)	18.1	(135.9)	1.9	(14.5)	18.3	(137.3)	1.4	(10.3)
Careless 3024	26.9	(201.5)	2.3	(17.0)	10.7	(80.4)	2.4	(17.9)	20.2	(150.9)	1.4	(10.6)	15.0	(112.1)	0.6	(4.4)
Careless (VT)	19.9	(130.7)	1.5	(9.7)	8.3	(55.2)	1.5	(10.1)	25.4	(190.3)	2.6	(19.4)	16.8	(125.8)	1.2	(8.8)
Ajax	19.4	(132.1)	1.7	(11.5)	10.3	(69.8)	1.9	(13.2)	19.4	(145.6)	2.3	(16.9)	16.1	(120.8)	1.3	(9.6)
Guy seedling	8.8	(44.7)	1.8	(9.1)	8.7	(43.8)	3.4	(17.2)	15.4	(115.1)	2.6	(19.2)	29.0	(217.4)	2.0	(15.1)
Mean	28.0	(196.1)	2.1	(14.7)	11.1	(77.2)	2.4	(16.7)	34.8	(260.9)	4.1	(31.2)	26.0	(192.8)	1.5	(11.3)
LSD^h^	8.25	(53.48)	0.49	(2.99)	2.27	(12.33)	0.48	(2.77)	16.34	(122.38)	2.10	(15.9)	8.40	(68.4)	0.50	(3.2)

a The results are presented in descending order according to the sum of flavonoids for FW.

b ProcB1 = procyanidin B1.

c ProcB2 = procyanidin B2.

d Q-3-rut = quercetin-3-rutinoside.

e Q-3-gluc = quercetin-3-glucoside.

f Isorh-3-rut = isorhamnetin-3-rutinoside.

g Isorh-3-gluc = isorhamnetin-3-glucoside.

h LSD = least significant difference at the 95% confidence level.

**Table 2:** Hydroxycinnamic acid derivative composition (mg kg^−1^) of the flesh/skin of 20 gooseberry cvs on a FW and DW basis (values inside brackets).^a^

Cultivar	Coumaroyl-hexose1		Coumaroyl-hexose2		Caffeoyl-hexose		Feruloyl-hexose1		Feruloyl-hexose2		Neochlorogenic acid	
Guy Seedling	45.5	(229.8)	15.3	(77.3)	39.9	(201.6)	2.3	(11.9)	11.8	(5.97)	10.4	(52.6)
Heart of Oak	41.5	(241.5)	18.2	(105.8)	33.4	(194.2)	1.5	(8.8)	8.2	(4.93)	7.7	(45.4)
Nailer	33.3	(198.8)	12.8	(76.5)	33.5	(199.8)	1.6	(9.9)	4.7	(2.88)	9.1	(54.5)
Jolly Amylers	38.2	(245.4)	16.7	(107.2)	24.0	(159.7)	1.7	(10.9)	7.0	(4.53)	3.8	(24.4)
Bedford Yellow	35.1	(244.5)	18.3	(127.9)	16.5	(114.7)	1.3	(8.9)	3.4	(2.44)	12.4	(87.0)
Rubuste Nool	32.3	(221.1)	13.3	(91.0)	24.9	(169.9)	2.8	(18.8)	6.9	(4.74)	4.2	(28.7)
Lord Elco	35.9	(286.0)	15.5	(123.7)	20.6	(164.6)	1.9	(15.2)	6.2	(4.99)	3.8	(30.3)
White Eagle	26.4	(168.8)	7.5	(48.0)	29.2	(186.4)	2.1	(13.7)	6.2	(3.97)	3.2	(20.8)
Careless VT	28.5	(218.9)	11.6	(88.9)	21.6	(166.0)	1.4	(11.0)	3.6	(2.76)	0.7	(5.4)
Scotch Red Rough	8.6	(48.5)	4.9	(27.3)	26.1	(145.6)	4.5	(25.0)	12.0	(6.68)	11.7	(66.8)
Mitre	25.6	(182.0)	9.3	(66.1)	20.8	(147.2)	1.4	(10.2)	3.4	(2.43)	0.8	(5.6)
Cousen’s Seedling	26.5	(212.1)	9.6	(76.1)	11.2	(89.4)	2.0	(16.0)	5.5	(4.46)	3.4	(27.9)
Careless (Kent)	27.1	(179.7)	10.3	(68.1)	14.3	(94.8)	1.5	(10.3)	3.9	(2.58)	0.7	(4.8)
Careless 3024	17.2	(129.0)	7.2	(54.2)	20.0	(150.1)	1.5	(11.2)	4.5	(3.38)	5.6	(41.8)
Lord Audley	19.3	(163.7)	6.1	(51.2)	15.0	(126.6)	1.3	(11.2)	3.4	(2.84)	8.8	(73.9)
Jubilee Careless	27.6	(186.2)	10.1	(68.0)	11.4	(76.3)	1.1	(7.5)	2.6	(1.78)	0.7	(4.8)
May Dulle	6.4	(45.7)	5.5	(39.5)	22.3	(158.8)	2.3	(16.1)	5.1	(3.64)	8.4	(69.1)
Victoria	14.5	(118.6)	6.9	(56.0)	17.0	(138.4)	1.0	(8.2)	3.8	(3.04)	5.1	(42.2)
Goutray’s Earliest	12.3	(107.2)	5.3	(46.7)	17.8	(155.5)	1.1	(9.6)	3.4	(2.95)	6.7	(58.1)
Ajax	17.2	(116.5)	5.8	(39.4)	13.5	(91.7)	1.4	(9.6)	4.0	(2.70)	3.9	(26.5)
Mean	26.0	(177.2)	10.5	(71.9)	21.7	(146.6)	1.8	(12.2)	5.5	(3.68)	5.6	(38.5)
LSD	5.31	(24.87)	2.59	(12.36)	5.75	(29.94)	0.51	(3.63)	0.98	(0.532)	1.61	(11.50)

a The results are presented in descending order according to the sum of hydroxycinnamic acid derivatives for FW.

Flavan-3-ols and dimeric procyanidins were present in gooseberries in small to moderate amounts with (+)-catechin being the predominant with an average concentration of 28.0 mg kg^−1^ (FW). These results are comparable with previous reports, with the exception of (+)-catechin which was in concentrations higher than that previously reported (Häkkinen et al., 1999b). Very few reports are available describing the levels of procyanidins in gooseberries, however, according to Wu et al. (2004), procyanidins and prodelphinidins are present in gooseberries mainly in polymeric form.

Of the phenolic acids, only hydroxycinnamic acid derivatives were detected in low to moderate amounts in the skin and flesh of gooseberries. The presence of neochlorogenic acid (*trans*-5-*O*-caffeoylquinic acid) was verified through the use of a commercial standard and accurate mass measurements. Several hydroxycinnamic acid glycosides were also tentatively identified with QToF/MS/MS, based on accurate mass measurements and the fragmentation pattern acquired with MS/MS in three different voltages (10, 20, 40 eV) which matched the fragmentation pattern obtained for the corresponding aglycons. The most abundant fragments for each compound are presented in Table 3. The sugar moiety was a hexose and the most abundant pseudo-molecular ion in positive mode was in the form of a [M − Na]^+^ adduct. The following glycosides were identified in gooseberry skin/flesh extracts: *p*-coumaric, caffeic, ferulic and sinapic acid hexosides (Table 3).

**Table 3:** Tentative identification of anthocyanins and hydroxycinnamic acid glycosides in gooseberry cvs with QToF/MS/MS.

Peak	Ret. time (min)		*m*/*z*	Fragments MS/MS	Tentative identification	Molecular formula
Anthocyanins						
3	7.13	[M+]	463.1303	301.0995	Peonidin-3-glucoside	C_22_H_23_O_11_
3	7.27	[M+]	419.1190	287.055	Cyanidin-3-xyloside	C_20_H_19_O_10_
4	7.42	[M+]	609.2129	301.0994	Peonidin-3-rutinoside	C_28_H_33_O_15_
6	10.11	[M+]	595.1447	287.055	Cyanidin-glycoside	
7	10.36	[M+]	595.1480	(287.0814, 147.0570, 163.1326)	Cyanidin-coumaroyl-glucopyranoside	C_30_H_27_O_13_
8	10.96	[M+]	609.1594	301.705	Peonidin-glycoside	C_28_H_33_O_15_

Ret. time (min)		*m*/*z*	Fragments MS/MS	Tentative identification	Molecular formula
Hydroxycinnamic acid derivatives					
5.15, 6.10	[M − Na]+	349.0893	(165.0545, 147.0570)	Coumaric acid hexoses	C_15_H_18_O_8_
4.20	[M − Na]+	365.0862	(181.0508, 163.0402, 145.0283, 135.0437, 117.0333, 89.0383)	Caffeic acid hexose	C_15_H_18_O_9_
4.86, 6.42	[M − Na]+	379.0999	(195.0651, 177.0546)	Ferulic acid hexoses	C_16_H_20_O_9_
5.62	[M − Na]+	409.1109	(225.076, 207.0652, 175.0391)	Sinapic acid hexose	C_17_H_22_O_10_

Among the hydroxycinnamic acid glycosides detected in gooseberry flesh/skin extracts, coumaric and caffeic acid glycosides were the most abundant, followed by ferulic acid glycosides (Table 2). Sinapic acid glycosides were also detected in trace amounts (Table 3). ‘Guy Seedling’ was the cv with the highest content in hydroxycinnamic acid glycosides with a sum of 114.8 mg kg^−1^ (FW), while ‘Scotch Red Rough’ cv which exhibited the highest flavonoid content among all cvs, had low levels of hydroxycinnamic acid glycosides (56.1 mg kg^−1^). The levels of hydroxycinnamic acid glycosides reported here are in good agreement with those reported in berries from other species in the *Grossulariaceae* family (Määttä-Riihinen et al., 2004). Neochlorogenic acid was present in small amounts in gooseberry flesh/skin with a mean concentration of 5.6 mg kg^−1^ (FW) in ‘Scotch Red Rough’. Its concentration was almost 2-fold higher in coloured varieties when compared to green varieties. This is the first report of neochlorogenic acid in gooseberries. The presence of several phenolic acids has been previously reported in the literature including protocatechuic acid, *p*-hydroxybenzoic acid, *p*-coumaric acid, ellagic acid, ferulic acid, chlorogenic acid, sinapic acid, caffeic acid, syringic acid, vanillic acid and gallic acid (Filipiak-Szok et al., 2012, Mattila et al., 2006, Russell et al., 2009). It is notable that none of the above phenolic acids were detected in free form in gooseberries in our study and no hydroxybenzoic acids were detected in the berries of the gooseberry cvs analysed. Similarly Määttä-Riihinen et al. (2004) reported the presence of hydroxycinnamic acids in gooseberries and other berry species in conjugated form, mainly as caffeoyl- and *p*-coumaroylglucosides.

#### 3.1.2 Phenolic profile of gooseberry seeds

Unlike the flesh/skin extracts which contained a variety of different phenolic compounds, the only type of phenolic compounds detected in gooseberry extracts of seeds were hydroxycinnamic acid derivatives, which were tentatively identified as glycosides of caffeic, coumaric, ferulic and sinapic acids as described in Section 3.1.1. Two distinct peaks with the same spectral characteristic attributed to coumaric acid glycosides and ferulic acid glycosides were present in all seed extracts, indicating the presence of two isomers of coumaric acid and ferulic acid glycosides (Table 3). Their concentrations are presented in Table 4. Gooseberry seeds were observed as being particularly high in phenolic acid glycosides, with concentrations being 10 times higher compared to the flesh/skin extracts. There was some variability among different varieties with the highest concentrations observed for ‘Heart of Oak’. The qualitative profile was similar to the flesh/skin extracts, with coumaric and caffeic acid glycosides being the most abundant, followed by ferulic and sinapic acid glycosides. No phenolic acids were present in free form in the seed extracts, as it was confirmed by comparison with the corresponding aglycon standards. It is also notable that neochlorogenic acid, which was detected in the flesh/skin extracts, was absent in gooseberry seeds. Other compounds detected in the gooseberry seed extracts included trace amounts of (+)-catechin and (−)-epicatechin, quercetin-3-rutinoside, isorhamnetin-3-rutinoside and cyanidin-3-rutinoside and cyaniding-3-glucoside in some red cvs.

**Table 4:** Concentration of hydroxycinnamic acid glycosides (g kg^−1^) in the seeds of 20 gooseberry cvs on a DW basis.^a^

Cultivar	Coumaroyl-hexose1	Coumaroyl-hexose2	Caffeoyl-hexose	Feruloyl-hexose1	Feruloyl-hexose2	Sinapoyl-hexose	ΣHCAD^b^
Heart of Oak	4.55	1.09	6.65	0.15	1.10	0.20	13.74
Guy seedling	2.26	0.64	4.19	0.12	0.64	0.19	8.04
Victoria	1.85	0.67	3.99	0.19	0.68	0.33	7.71
Careless VT	2.21	0.75	3.03	0.15	0.76	0.31	7.21
Nailer	1.77	0.64	3.33	0.12	0.65	0.33	6.84
Mitre	1.81	0.65	3.26	0.15	0.66	0.28	6.81
Jolly Amylers	1.70	0.57	3.23	0.12	0.58	0.37	6.57
Bedford Yellow	1.97	0.76	1.74	0.17	0.76	0.24	5.64
May Dulle	1.35	0.46	2.47	0.15	0.47	0.18	5.08
Ajax	0.91	0.35	2.89	0.12	0.36	0.30	4.93
Jubilee Careless	1.52	0.51	1.99	0.13	0.51	0.25	4.91
Careless 3024	1.29	0.50	1.95	0.14	0.50	0.31	4.69
Careless (Kent)	1.55	0.49	1.82	0.11	0.49	0.17	4.63
Cousen’s Seedling	1.53	0.45	1.75	0.16	0.45	0.29	4.63
White Eagle	1.14	0.31	2.30	0.10	0.32	0.32	4.49
Goutray’s Earliest	0.82	0.33	2.48	0.13	0.33	0.20	4.29
Rubuste Nool	0.98	0.28	1.81	0.08	0.28	0.25	3.68
Lord Elco	0.99	0.30	1.60	0.10	0.30	0.31	3.60
Lord Audley	0.71	0.24	1.75	0.11	0.24	0.15	3.20
Scotch Red Rough	0.61	0.21	1.72	0.16	0.21	0.05	2.96
Mean	1.58	0.51	2.70	0.13	0.51	0.25	5.68
LSD	0.356	0.15	0.917	0.036	0.151	0.077	1.393

a The results are presented in descending order according to the sum of hydroxycinnamic acid derivatives for DW.

b ΣHCAD = Sum of hydroxycinnamic acid derivatives.

To our knowledge no other reports exist on the phenolic composition of gooseberry seeds, but some comparisons can be made with other *Ribes* species. Gođevac et al. (2012) have tentatively identified 35 compounds in black, red and white currant seed extracts, including phenolic acids derivatives such as sinapoyl glucoside and chlorogenic acid and several phenolic acids in free form, small amounts of flavonols and flavonol glycosides as well as two nitrile containing derivatives of *p*-coumaric and ferulic acid. In contrast Bakowska-Barczak et al. (2009), have reported flavonols as the main phenolic group present in blackcurrant seed residue in the form of kaempferol, myricetin and quercetin glycosides. The same authors also reported the presence of amounts of *p*-coumaric acid along with small amounts of *p*-coumaroyl-glucoside in the seeds (0.487 g kg^−1^ and 0.179 g kg^−1^, respectively, for DW), although the presence of the aglycon was possibly a degradation product (Bakowska-Barczak et al., 2009). Lu et al. (2002) isolated six glucosides of quercetin, kaempferol and myricetin from blackcurrant seeds along with two nitrile containing derivatives of *p*-coumaric acid. Furthermore, Lu and Foo (2003) isolated and characterised by ^13^C NMR two phenolic acid conjugates namely 1-*p*-coumaroyl-β-d-glucopyranoside and 1-cinnamoyl-β-d-glucopyranoside from blackcurrant seeds along with several anthocyanins and flavonoids.

The presence of phenolic acid glycosides has also been reported in the genus *Fragaria*, with several hexoses of coumaric, ferulic, caffeic and sinapic acids reported primarily in the strawberry receptacle and in small amounts in the achenes (Fait et al., 2008). These differences in the diversity of compounds and their concentrations could be attributed to differences among species, cvs, extraction and detection methods.

#### 3.1.3 Characterization and quantification of anthocyanins

A total of eight anthocyanins were detected in the pink and red gooseberry varieties. Cyanidin-3-rutinoside and cyanidin-3-glucoside were identified by comparison with authentic standards. The other anthocyanins were tentatively identified based on their accurate mass and the fragmentation pattern acquired with MS/MS in different voltages. (Table 3). The major daughter ion produced was the aglycon after the loss of the sugar moiety. Peak 3 corresponded to peonidin-3-glucoside, with small amounts of cyanidin-3-xyloside, also co-eluting under the same peak as revealed by the mass profile obtained with Q-ToF/MS/MS. Peak 4 was tentatively identified as peonidin-3-rutinoside. Peaks 6 and 7 had accurate mass at *m/z* 595.1447, and 595.1480, respectively, and after fragmentation they both yielded a major daughter ion with *m/z* 287.055 which was attributed to cyanidin aglycon. Peak **7** additionally had a daughter ion with *m/z* 147.0570 which is a characteristic fragment of *p*-coumaric acid. Jordheim et al. (2007), have characterised two isomers of cyanidin-3-*O*-*p*-coumaroyl-glucopyranoside in gooseberries by HPLC-DAD and NMR spectroscopy. Peak 7, furthermore had the same UV/Vis spectra (Fig. S1) as cyanidin-3-*O*-β-(6′′-*E*-*p*-coumaroylglucopyranoside) as reported by Jordheim et al. (2007). Therefore it was tentatively identified as cyanidin-*p*-coumaroyl-glucopyranoside. Peak 8 exhibited a similar mass profile as peak 4 and it was tentatively identified as peonidin-3-glycoside. Peak 5 was present in very low amounts and it was not possible to acquire a clear LC/MS profile.

The anthocyanin concentrations in pigmented gooseberry cvs are presented in Table 5. Cyanidin-3-rutinoside (pigment 2) was the predominant anthocyanin identified in all the cvs followed by cyanidin-3-glucoside (pigment 1) and pigment 7. ‘Scotch Red Rough’ exhibited the highest total anthocyanin content with a sum of 265.8 mg kg^−1^ (FW), which is comparable with the levels of anthocyanins reported for other red gooseberry cvs and similar to the levels found in red currants (Djordjevic et al., 2010, Jordheim et al., 2007, Määttä-Riihinen et al., 2004).

**Table 5:** Concentration of anthocyanins (mg kg^−1^) in 20 gooseberry cvs on a FW and DW basis (values inside brackets). The results are presented in descending order according to the sum of anthocyanins per FW.^a^

Cultivar	**1**		**2**		**3**		**4**		**5**		**6**		**7**		**8**	
Scotch Red Rough	48.3^d^	(267.5)	128.8^h^	(714.6)	3.3^c^	(18.5)	14.5^c^	(82.9)	6.0b^a^	(31.9)	5.7^c^	(31.7)	57.3^e^	(319.8)	1.9^a^	(10.9)
May Dulle	33.6^d^	(239.0)	36.3^g^	(256.6)	2.0^b^	(14.0)	2.9^b^	(20.6)	1.5^b^	(10.7)	2.6^b^	(18.2)	21.1^d^	(149.9)	0.4^b^	(3.1)
Careless 3024	8.2^ab^	(61.6)	19.5^f^	(146.4)	t^b^		0.8^a^	(6.2)	t		0.8^a^	(5.7)	4.6^c^	(34.4)	t	
Victoria	9.4^b^	(83.9)	13.5^c^	(109.7)	nd^c^		t		t		0.5^a^	(4.4)	2.8^a^	(23.1)	nd	
Goutray’s Earliest	6.1^ab^	(53.6)	8.3^abc^	(72.0)	0.8^a^	(6.9)	0.9a	(7.7)	0.6c	(5.0)	t		3.0^a^	(26.1)	t	
Ajax	5.3^ac^	(36.1)	9.3^bc^	(63.1)	t		1.1a	(7.7)	t		t		2.1^a^	(14.2)	nd	
Lord Audley	3.6^c^	(30.5)	4.8^a^	(40.9)	t		t		t		nd		1.4^b^	(11.8)	nd	
Heart of Oak	1.0^e^	(6.2)	5.4^ab^	(31.7)	nd		t		nd		t		t		nd	
Bedford Yellow	t		1.8^e^	(12.2)	t		t		t		t		t		t	
Nailer	t		1.2^de^	(7.3)	nd		t		nd		nd		t		t	
Jolly Amylers	t		0.6^d^	(4.3)	nd		t		nd		t		t		nd	
Mean	14.6	(9.73)	20.9	(132.6)	2.0	(13.2)	4.0	(25.0)	2.7	(15.9)	2.4	(15.0)	13.2	(82.8)	1.2	(7.0)
LSD					0.58	(4.05)	1.17	(2.75)	0.38	(2.76)	0.68	(4.22)			0.69	(4.59)

a The cultivars for which the levels of anthocyanins were below the detection limit (DL) or quantification limit (QL) have been omitted from the table.

b t = traces.

c nd = not detected. Means within the same column with no letters in common are significantly different. Data for which LSD is not provided have been log transformed and the significant differences are based on the analysis of variance (ANOVA) for the logarithmic data.

### 3.2 Presence of non-structural carbohydrates

The concentration of total sugars on a FW basis, ranged between 31.4 g kg^−1^ to 80.2 g kg^−1^ (Table 6), which is consistent with previous reports (Mikulic-Petkovsek et al., 2012b, Viljakainen et al., 2002). Fructose and glucose were the predominant sugars in all cvs accounting for ∼49% and ∼40.5%, respectively, of total sugars. The mean ratio of sucrose was ∼10% of the total sugars in gooseberry cvs. These results are in agreement with Giné Bordonaba and Terry (2008) who reported the same ratios of sugars in 17 UK-grown blackcurrant cvs, indicating a similar sugar profile of different species belonging to the *Grossulariaceae*. The ratio of fructose to glucose reported here varied from 0.9 to 1.3 and is within the range reported in red and blackcurrants (Milivojevic et al., 2012). The relative proportion of individual sugars in fruits can influence the taste properties such as sweetness (Milivojevic et al., 2012).

**Table 6:** Concentration of non-structural carbohydrates (g kg^−1^) in 20 gooseberry cultivars on a FW and DW basis (values inside brackets).^a^

Cultivar	Fructose		Glucose			Sucrose			Total		
Guy seedling	37.5^h^	(189.8)	31.7^f^		(160.6)	10.9^e^		(55.0)	80.2^g^		(405.4)
Jubilee Careless	34.1^gh^	(228.4)	29.0^ef^		(193.5)	9.4 ^de^		(61.4)	72.5^fg^		(483.3)
Ajax	33.2^fgh^	(225.2)	28.2^def^		(191.7)	6.4^cd^		(43.5)	67.8^fg^		(460.3)
Scotch Red Rough	30.9^fgh^	(173.9)	24.5^cdef^		(138.2)	11.5^e^		(63.4)	66.9^fg^		(375.5)
Careless (Kent)	32.1^fgh^	(212.9)	28.0^def^		(185.9)	6.3^cd^		(41.8)	66.4^fg^		(440.6)
Rubuste Nool	32.5^fgh^	(218.2)	27.4^cdef^		(183.8)	6.0^cd^		(44.0)	65.9^fg^		(442.4)
White Eagle	30.8^fgh^	(196.8)	27.7^cdef^		(176.3)	6.6^cd^		(41.9)	65.1^fg^		(415.0)
Careless VT	31.6^fgh^	(242.4)	27.3^cdef^		(209.3)	4.5^bc^		(34.8)	63.5^fg^		(486.4)
Bedford Yellow	31.0^fgh^	(218.2)	25.7^cdef^		(180.7)	5.7^c^		(40.0)	62.4^fg^		(439.0)
Nailer	27.8^efgh^	(166.1)	22.1^bcde^		(132.1)	10.8^e^		(64.0)	60.7^f^		(362.2)
Heart of Oak	24.7^cdef^	(143.5)	20.3^bcd^		(118.1)	14.3^e^		(82.5)	59.3^f^		(344.2)
Cousen's Seedling	29.3^efgh^	(235.4)	24.9^cdef^		(200.2)	3.1^ab^		(25.0)	57.4^ef^		(460.7)
Mitre	27.3^efg^	(195.0)	23.3^cdef^		(166.4)	5.3^c^		(37.7)	55.9^def^		(399.1)
May Dulle	26.1^defg^	(185.7)	22.0^bcde^		(156.3)	4.6^c^		(32.8)	52.7^cdef^		(374.8)
Jolly Amylers	25.0^bcdef^	(159.4)	20.7^bc^		(131.9)	5.5^c^		(35.2)	51.3^bcdef^		(326.6)
Victoria	21.9^abcde^	(178.8)	16.4^ab^		(133.9)	2.9^a^		(23.5)	41.2^abcde^		(336.2)
Lord Elco	19.5^abcd^	(155.7)	16.3^ab^		(129.0)	2.9^a^		(22.8)	38.6^abcd^		(308.5)
Goutray's Earliest	19.3^abc^	(168.5)	14.2^a^		(123.4)	3.1^a^		(27.2)	36.6^abc^		(319.0)
Lord Audley	17.8^ab^	(153.0)	14.0^a^		(118.4)	2.8^a^		(23.9)	34.6^ab^		(292.6)
Careless 3024	16.6^a^	(124.1)	12.0^a^		(90.0)	2.8^a^		(21.2)	31.4^a^		(235.3)
Mean	27.5	(188.4)	22.8		(156.0)	6.3		(40.9)	56.5		(385.4)

a The results are presented in descending order according to total sugar content for FW. Means within the same column with no letters in common are significantly different. Results are based on the analysis of variance (ANOVA) for the log transformed data for all the sugars.

### 3.3 Total ascorbic acid

Ascorbic acid was determined as total ascorbic acid which comprised ascorbic acid and its oxidised form (dehydroascorbic acid). It was detected as a single peak at 3.1 min. The vitamin C content varied from 0.38 g kg^−1^ in ‘White Eagle’ to 0.85 g kg^−1^ (FW) in ‘Ajax’ (Table 7). These concentrations are up to 3-fold higher than previous reported for gooseberries (Häkkinen et al., 1999b, Pantelidis et al., 2007, USDA, 2014), although they are similar to the concentrations reported in blackcurrants (Djordjevic et al., 2010), red currant, strawberry and lingonberry (Skrede et al., 2012) and within the range found in common berries (<1 g kg^−1^ FW) (Giné Bordonaba and Terry, 2011). The differences between these and earlier studies could reflect differences in sample preparation and analytical methods or more importantly differences among varieties.

**Table 7:** Concentration of total ascorbic acid (g kg^−1^) in 20 gooseberry cvs on a FW basis.^a^

Cultivar	Total ascorbic acid
Ajax	0.85
Careless VT	0.83
Jubilee Careless	0.81
Lord Elco	0.74
Careless (Kent)	0.74
Nailer	0.68
Scotch red rough	0.64
Heart of Oak	0.60
Victoria	0.56
Bedford yellow	0.55
Guy Seedling	0.54
Mitre	0.53
Cousen’s Seedling	0.53
Careless 3024	0.52
May Dulle	0.51
Lord Audley	0.48
Robuste Nool	0.45
Goutrey Earliest	0.44
Jolly Amylers	0.43
White Eagle	0.38
Mean	0.59
LSD	0.049

a The results are presented in descending order according to total ascorbic acid content.

### 3.4 Postharvest treatment of gooseberries

The two cvs selected for the storage trial in the following year, included the red cv ‘Scotch Red Rough’ which exhibited the most diverse profile of phenolics and had a high concentration of anthocyanins as well as the green cv ‘Careless (Kent)’ which is a popular commercial variety of gooseberry. For both cvs (per DW), the phenolic and sugar content at harvest point was compared between the two successive years. The composition of non-structural sugars between the two seasons was similar, with only sucrose in ‘Careless Kent’ dropping from 41.8 g kg^−1^ in the first year to 26.5 g kg^−1^ in the second (*p* < 0.05) (Table S1). Overall, the profile of phenolics was similar for both years; the most notable difference was the 2-fold drop in anthocyanin content in ‘Scotch Red Rough’ during the second year from 1477.8 mg kg^−1^ to 749.5 mg kg^−1^ (Table S1).

#### 3.4.1 Biochemical and physiology changes during storage

In the present study the changes in major phenolic compounds, non-structural sugars, colour and ethylene emission were recorded for both cvs, in order to determine the biochemical and physiology changes in gooseberry fruit during air storage and the effect of external ethylene exposure. The water content was also monitored for both cvs throughout the storage period. ‘Careless Kent’ had a consistently higher water content compared to ‘Scotch Red Rough’ with average values at baseline of 87.7% and 81.8%, respectively. The results indicated a marginal water loss of ≤2% for both cvs, irrespective of treatment, which occurred after 7 days of storage (Table S2). Also there was no leakage recorded in any of the cvs.

#### 3.4.2 Ethylene

**Fig. 2:**
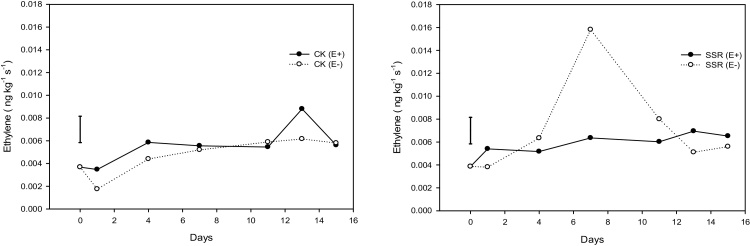
Ethylene emission recorded during the storage period for the two gooseberry cvs, (E +) = ethylene treated samples, E(−) = control samples, CK = ‘Careless (Kent)’, SSR = ‘Scotch Red Rough’. The error bars represent the least significant difference (LSD) at 95% confidence level.

The change in ethylene production rates for treated and control samples is presented in Fig. 2. Ethylene emission remained at low levels throughout the storage period with a total mean of 5.8 × 10^−3^ ng kg^−1^ s^−1^, which is significantly lower compared to other soft fruits such as blackberries and raspberries (0.035–0.35 ng kg^−1^ s^−1^ at 5 °C) (Mitcham et al., http://postharvest.ucdavis.edu/PFfruits/Bushberries/). An increasing trend was observed for the red cv with the control samples showing a sharp increase towards the middle of the storage period, but subsequently fell in the same levels as the treated samples. Ethylene emission levels remained relatively stable for the green variety throughout the storage period.

#### 3.4.3 Phenolic compounds

The biochemical results, on a dry weight basis, showed that flavonol glycosides, which represent the major group of phenolic compounds in gooseberries, remained relatively stable for both treated and control samples throughout the storage period. ‘Scotch Red Rough’ had consistently higher concentrations of flavonol glycosides during the trial, as it is characteristic of the cv (Table S3).

**Fig. 3:**
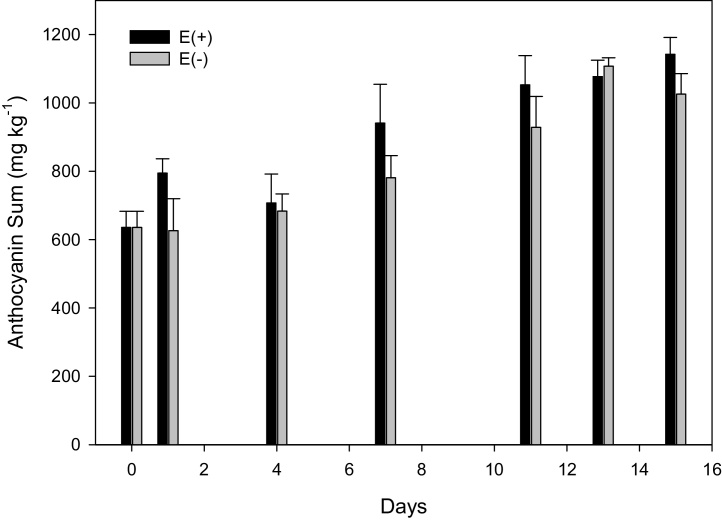
Concentration of total anthocyanins on a DW basis, in ‘Scotch Red Rough’ during the storage period. E(+) = Ethylene treated samples, E(−) = control. The error bars represent the standard error (SE) of the mean.

Anthocyanins on the other hand exhibited an increasing trend in ‘Scotch Red Rough’ over time irrespective of treatment (Fig. 3). A marginal increase observed for the ethylene treated samples was not recorded towards the end of the storage period with both treated and control samples, reaching the same anthocyanin concentrations. This could indicate an initial positive effect of exogenous ethylene on the accumulation of anthocyanins which was counteracted by the sudden increase in ethylene production observed in the control samples towards the middle of the storage period. Other non-climacteric fruits, such as grapes, have exhibited a clear response to postharvest ethylene treatment with anthocyanins positively affected at the end of the treatment period (Becatti et al., 2010, Becatti et al., 2014). These differences could be due to species differences and also differences in the treatment protocols applied. Grape berries were exposed to very high ethylene concentrations (1000 mg L^−1^) and for a longer period of time (36 h), which could explain the acute metabolic responses to ethylene.

On the other hand, the accumulation of anthocyanins under air-storage, observed for the red gooseberry cv, seems to be a common trend among berry species, Kim et al. (2015), recently have demonstrated an increase in anthocyanin content in blackberries during storage at 1 °C, which was more pronounced if the berries were transferred to 20 °C towards the end of the storage period.

Earlier reports, suggest that gooseberry exposure to ethylene, can cause some yellowing/browning of the skin (Cantwell, 2002). In the current study, colour measurements revealed a decrease in L* and C* values over time, for both treated and control gooseberries (Table S2), which was not associated with any perceived colour deterioration. The decrease in L* values could be attributed to several reasons including the observed accumulation of anthocyanin and perhaps gradual water loss. The change in C* values, early in the storage period, may be related to an initial response to exogenous ethylene for the treated samples and the increase in ethylene emission for the control samples, which was further enhanced by water loss later in the storage period.

#### 3.4.4 Non-structural carbohydrates

**Fig. 4:**
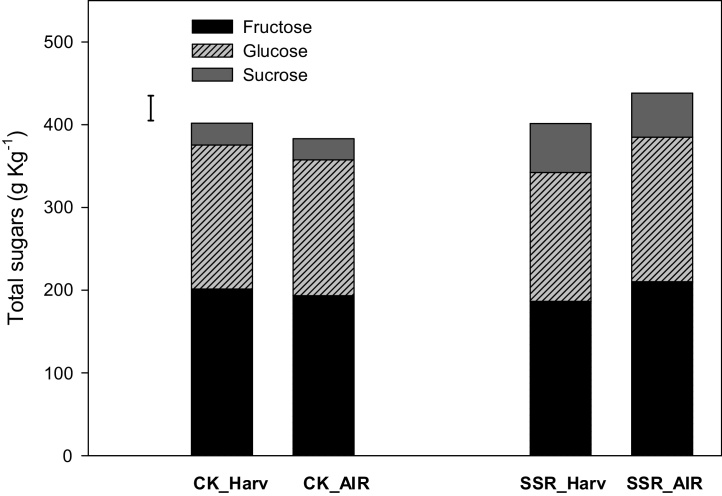
Average concentrations of non-structural carbohydrates on a DW basis, before and during storage for the two gooseberry cvs. CK_Harv = ‘Careless (Kent)’ at harvest, CK_AIR = ‘Careless (Kent)’ under air storage, SSR_Harv = ‘Scotch Red Rough’ at harvest, SSR_AIR = ‘Scotch Red Rough’ under air storage.

The concentrations of non-structural carbohydrates, for both cvs examined, remained relatively stable for both treated and control samples. The main difference in sugar composition between the two cvs was in the levels of sucrose, which were particularly elevated in ‘Scotch Red Rough’ throughout the storage period. The average concentration of total sugars for the two cvs, at baseline and during the storage period, is shown in Fig. 4. A previous study on ‘Achilles’ gooseberries stored under air and different CA conditions, showed that total soluble solids, remained stable for 7 weeks (Harb and Streif, 2004).

The biochemistry and physiology results overall, reveal that gooseberries did not show any clear response to the application of exogenous ethylene and are in broad agreement with previous reports classifying gooseberries as fruits with low sensitivity to ethylene (Cantwell, 2002).

## 4 Conclusions

The present study reveals the potential for the use of gooseberries as a rich source of bioactive compounds and demonstrates the distinguishing variability across cultivars and tissue types. Furthermore, it enhances our understanding of their postharvest behaviour especially in relation to their response to exogenous ethylene and the preservation of their nutritional quality under cold storage.
