# Supplementary material for: Tissue biochemical diversity of 20 gooseberry cultivars and the effect of ethylene supplementation on postharvest life
Source: Postharvest Biol Technol. 2016 Jul;117:141–51. doi: 10.1016/j.postharvbio.2016.02.008 (PMC6472321; doi:10.1016/j.postharvbio.2016.02.008)
Supplement: Supplementary file 1 [file mmc1.docx]

**Table S1**. Yearly variation of phenolic compounds and non-structural carbohydrates on a dry weight (DW) basis, for two gooseberry cvs.^a^

| **Careless (Kent)** | | | | | | | | | |
| --- | --- | --- | --- | --- | --- | --- | --- | --- | --- |
|  | Fructose | Glucose | Sucrose | Total Sugars | Q-3-rut^b^ | Q-3-gluc^c^ | Isorh-3-rut^d^ | Isorh-3-gluc^e^ | Total Flavonols |
| Year1 | 212.9^a^ | 185.9^a^ | 41.8^a^ | 440.6^a^ | 322.8^a^ | 39.9^a^ | 215.0^a^ | 17.0^a^ | 592.1^a^ |
| Year2 | 201.4^a^ | 174.0^a^ | 26.5^b^ | 402.0^a^ | 482.1^a^ | 57.3^a^ | 224.8^a^ | 7.5^b^ | 771.6^a^ |
| **Scotch Red Rough** | | | | | | | | | |
|  | Fructose | Glucose | Sucrose | Total Sugars | Q-3-rut | Q-3-gluc | Isorh-3-rut | Isorh-3-gluc | Total Flavonols |
| Year1 | 173.9^a^ | 138.2^a^ | 63.4^a^ | 375.6^a^ | 680.1^a^ | 102.7^a^ | 553.9^a^ | 20.4^a^ | 1357.1^a^ |
| Year2 | 186.5^a^ | 155.7^a^ | 59.1^a^ | 401.3^a^ | 710.3^a^ | 97.0^a^ | 659.9^a^ | 20.1^a^ | 1487.3^a^ |
| **Scotch Red Rough** | | | | | | | | | |
|  | **1** | **2** | **3** | **4** | **5** | **6** | **7** | **8** | Total Anthocyanins |
| Year1 | 267.5^a^ | 714.6^a^ | 18.5^a^ | 85.0^a^ | 25.9^a^ | 31.7^a^ | 319.8^a^ | 10.9^a^ | 1473.9^a^ |
| Year2 | 113.9^b^ | 357.5^b^ | 12.3^b^ | 52.0^b^ | 21.5^a^ | 18.5^b^ | 162.5^b^ | 11.3^a^ | 749.5^b^ |

^a^ The concentration of non-structural carbohydrates is expressed in g kg^-1^ (DW) and the concentration of phenolics in mg kg^-1^ (DW). Means within the same column with no letters in common are significantly different.

^b^ Q-3-rut= quercetin-3-rutinoside.

^c^ Q-3-gluc=quercetin-3-glucoside.

^d^ Isorh-3-rut=isorhamnetin-3-rutinoside.

^e^ Isorh-3-gluc=isorhamnetin-3-glucoside.
