# Supplementary material for: Tissue biochemical diversity of 20 gooseberry cultivars and the effect of ethylene supplementation on postharvest life
Source: Postharvest Biol Technol. 2016 Jul;117:141–51. doi: 10.1016/j.postharvbio.2016.02.008 (PMC6472321; doi:10.1016/j.postharvbio.2016.02.008)
Supplement: Supplementary file 2 [file mmc2.docx]

**Table S2.** Changes in water content and colour properties in ‘Careless (Kent)’ and ‘Scotch Red Rough’, over the storage period.

| **‘Careless (Kent)’** | | | | | | | | | | | | |
| --- | --- | --- | --- | --- | --- | --- | --- | --- | --- | --- | --- | --- |
| %Water Content | | | | Chroma | | | Lightness | | | Hue | | |
| Days | E(+)^a^ | | E(-)^b^ | E(+) | | E(-) | E(+) | | E(-) | E(+) | | E(-) |
| 0 | 87.7 | | 87.7 | 23.6 | | 23.6 | 53.6 | | 53.6 | 106.0 | | 106.0 |
| 1 | 87.8 | | 87.8 | 19.4 | | 22.2 | 53.1 | | 53.1 | 105.3 | | 106.4 |
| 4 | 87.7 | | 87.2 | 24.4 | | 25.2 | 52.6 | | 54.3 | 108.0 | | 109.4 |
| 7 | 86.8 | | 87.1 | 24.7 | | 24.5 | 52.8 | | 51.6 | 106.7 | | 108.5 |
| 11 | 86.8 | | 87.1 | 24.3 | | 23.9 | 50.0 | | 50.6 | 108.4 | | 108.6 |
| 13 | 86.6 | | 86.8 | 25.7 | | 24.8 | 50.9 | | 50.2 | 107.8 | | 110.7 |
| 15 | 85.8 | | 86.9 | 27.0 | | 24.3 | 52.2 | | 50.5 | 106.4 | | 108.6 |
| **‘Scotch Red Rough’** | | | | | | | | | | | | |
|  | %Water Content | | | | Chroma | | | Lightness | | | Hue | |
| Days | E(+) | | E(-) | E(+) | | E(-) | E(+) | | E(-) | E(+) | | E(-) |
| 0 | 81.8 | 81.8 | | 26.2 | | 26.2 | 33.5 | | 33.5 | 21.7 | | 21.7 |
| 1 | 81.8 | 81.7 | | 22.4 | | 24.2 | 34.3 | | 27.3 | 16.3 | | 15.7 |
| 4 | 81.8 | 81.5 | | 19.2 | | 19.2 | 28.6 | | 30.0 | 21.8 | | 23.6 |
| 7 | 80.5 | 80.5 | | 17.3 | | 19.3 | 27.8 | | 27.1 | 21.3 | | 22.0 |
| 11 | 80.6 | 80.9 | | 18.9 | | 20.7 | 28.4 | | 27.2 | 21.1 | | 22.7 |
| 13 | 78.8 | 79.6 | | 19.3 | | 20.7 | 24.1 | | 25.6 | 19.8 | | 20.8 |
| 15 | 80.4 | 79.7 | | 18.2 | | 19.1 | 24.8 | | 26.1 | 19.9 | | 21.0 |
| LSD^c^ | |  | | 2.35 | | | 2.24 | | |  | |  |

^a^ E(+) = ethylene treated samples.

^b^ E(-) = control.

^c^ LSD = least significant difference at the 95% confidence level. Where there is no LSD displayed there are no statistically significant differences.
