# Supplementary material for: Tissue biochemical diversity of 20 gooseberry cultivars and the effect of ethylene supplementation on postharvest life
Source: Postharvest Biol Technol. 2016 Jul;117:141–51. doi: 10.1016/j.postharvbio.2016.02.008 (PMC6472321; doi:10.1016/j.postharvbio.2016.02.008)
Supplement: Supplementary file 3 [file mmc3.docx]

**Table S3**. Concentration of flavonol glycosides (mg kg^-1^) during the storage period for the two gooseberry cvs expressed on a dry weight (DW) basis.^a^

| **Careless (Kent)** | | | | | | | | |
| --- | --- | --- | --- | --- | --- | --- | --- | --- |
|  | Q-3-rut^b^ | | Q-3-gluc^c^ | | Isorh-3-rut^d^ | | Isorh-3-gluc^e^ | |
| Days | (E+)^f^ | (E-)^g^ | (E+) | (E-) | (E+) | (E-) | (E+) | (E-) |
| 0 | 482.1 | 482.1 | 57.3 | 57.3 | 224.8 | 224.8 | 7.5 | 7.5 |
| 1 | 483.2 | 549.6 | 55.6 | 64.9 | 221.2 | 255.2 | 7.7 | 9.4 |
| 4 | 509.8 | 454.3 | 58.4 | 54.7 | 244.2 | 221.6 | 10.7 | 7.7 |
| 7 | 742.9 | 660.4 | 90.3 | 77.9 | 287.2 | 317.7 | 12.1 | 13.9 |
| 11 | 419.2 | 541.5 | 48.1 | 62.0 | 221.1 | 263.7 | 8.1 | 10.0 |
| 13 | 540.8 | 477.5 | 58.5 | 51.8 | 259.9 | 226.6 | 9.7 | 7.6 |
| 15 | 591.3 | 461.4 | 89.6 | 52.9 | 332.5 | 245.4 | 13.7 | 9.4 |
| **Scotch Red Rough** | | | | | | | | |
|  | Q-3-rut | | Q-3-gluc | | Isorh-3-rut | | Isorh-3-gluc | |
| Days | (E+) | (E-) | (E+) | (E-) | (E+) | (E-) | (E+) | (E-) |
| 0 | 710.3 | 710.3 | 97.0 | 97.0 | 659.9 | 659.9 | 20.1 | 20.1 |
| 1 | 732.3 | 690.0 | 104.6 | 90.6 | 678.7 | 615.1 | 19.7 | 18.0 |
| 4 | 538.4 | 607.1 | 78.5 | 85.8 | 496.3 | 580.7 | 14.3 | 16.6 |
| 7 | 750.1 | 699.9 | 104.7 | 92.4 | 702.8 | 650.4 | 19.2 | 17.4 |
| 11 | 606.3 | 763.3 | 95.7 | 102.3 | 699.3 | 679.4 | 21.9 | 19.0 |
| 13 | 784.4 | 624.9 | 114.6 | 83.7 | 688.9 | 461.7 | 19.7 | 14.2 |
| 15 | 788.2 | 708.3 | 112.5 | 103.6 | 697.0 | 643.8 | 23.3 | 19.5 |
|  |  |  |  |  |  |  |  |  |

^a^ Analysis of variance (ANOVA) indicated that the concentrations of flavonol glycosides during storage, did not differ significantly from the baseline.

^b^ Q-3-rut = quercetin-3-rutinoside.

^c^ Q-3-gluc = quercetin-3-glucoside.

^d^ Isorh-3-rut = isorhamnetin-3-rutinoside.

^e^ Isorh-3-gluc = isorhamnetin-3-glucoside.

^f^ E(+) = ethylene treated samples.

^g^ E(-) = control.
