# Supplementary material for: Tissue biochemical diversity of 20 gooseberry cultivars and the effect of ethylene supplementation on postharvest life
Source: Postharvest Biol Technol. 2016 Jul;117:141–51. doi: 10.1016/j.postharvbio.2016.02.008 (PMC6472321; doi:10.1016/j.postharvbio.2016.02.008)
Supplement: Supplementary file 4 [file mmc4.docx]

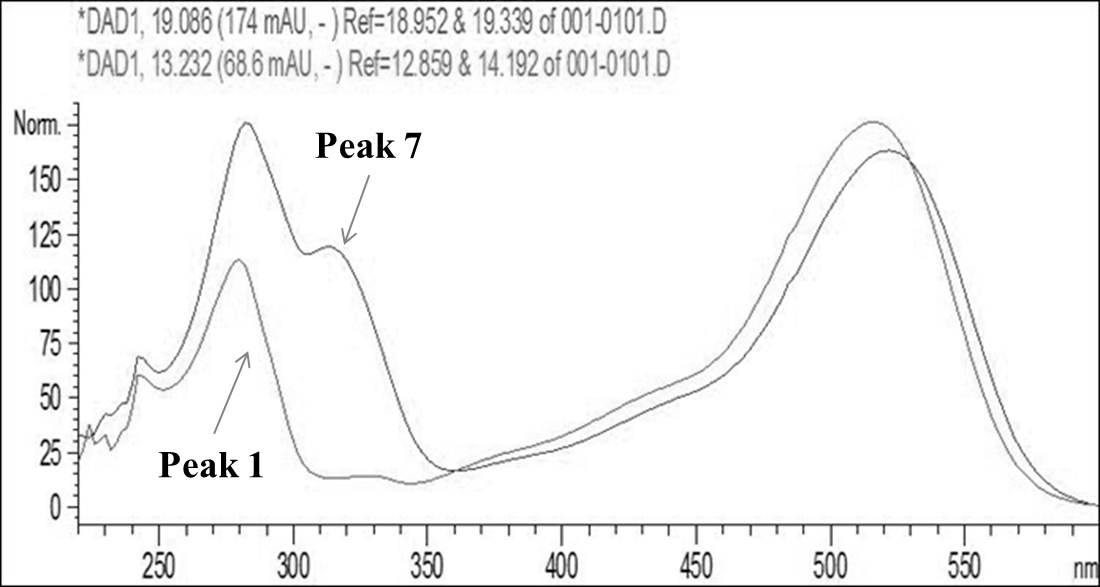


**Figure S1.** UV/Vis spectra of cyanidin-3-glucoside (Peak **1**) and Peak **7** isolated at 520nm from ‘Scotch Red Rough’ phenolic extract (skin/flesh).
